# Menstrual and sexual functions in female patients after sleeve gastrectomy due to obesity: obesity and sexual function

**DOI:** 10.1007/s00404-025-07966-z

**Published:** 2025-02-05

**Authors:** Hayal Uzelli Şimşek, Ece Nur Varol, Sertaç Ata Güler, Turgay Şimşek, Enes Şahin, Nuh Zafer Cantürk

**Affiliations:** 1 Department of Obstetrics and Gynecology, Kocaeli University School of Medicine, Umuttepe / Uctepeler 41000, Kocaeli, Turkey; 2 Department of General Surgery, Kocaeli University School of Medicine, Kocaeli, Turkey

**Keywords:** Body mass index (BMI), Excess weight loss (EWL), Female sexual function scale (FSFI), Menstrual regularity

## Abstract

**Purpose:**

Obesity is a systemic condition that is increasingly common. Obesity negatively affects sexual function and menstrual regularity. Therefore, losing excess weight is important for women's sexual and menstrual health.

**Methods:**

The change in body mass index (BMI) and and excess weight loss (EWL) of sexually active female patients who underwent laparoscopic sleeve gastrectomy (LSG) were evaluated by a general surgeon. The preoperative and postoperative periods were compared by a gynecologist using the survey method menstrual patterns, dysmenorrhea complaints, if any, and sexual function with the Female Sexual Function Scale (FSFI).

**Results:**

The study included 55 patients with a mean ± standard deviation BMI on the day of the operation of 45.32 ± 5.82 kg/m2. In the first postoperative year, the mean BMI significantly reduced to 27.88 ± 1.99 (*p* < 0.001). The mean percentage of EWL at the end of 1 year was 73.09 ± 19.74 after LSG. The median (range) preoperative FSFI score of the patients was 26.30 (22.70–27.70). One year after LSG, the median FSFI score significantly improved to 34.50 (30.20–35.30) (*p* < 0.001). Compared to the period before surgery, the frequency of sexual intercourse increased from two-to-three times a week (*p* < 0.001).

**Conclusion:**

Women's sexual desires are a fundamental human right and contribute to female well-being. Thus, it is important to treat sexual dysfunction. The results of the present study demonstrate a significant improvement in sexual dysfunctions after LSG. LSG was an effective procedure that may be recommended to obese women with sexual dysfunction and menstrual problems.

**Graphical abstract:**

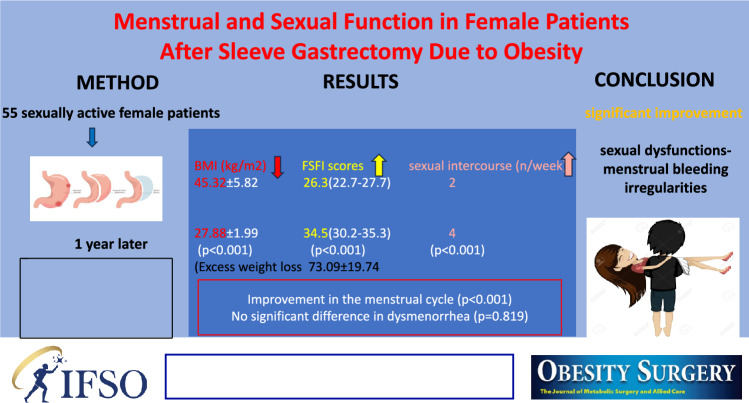

## What does this study add to the clinical work

**Table:** 

An improvement in menstrual bleeding irregularities and more importantly, an improvement in sexual function was detected with losing excess weight. If there are patients in gynecology clinics who have menstrual and/or sexual dysfunction, they should be encouraged to lose excess weight and the necessary consultations should be provided.	

## Introduction

Obesity is defined as a body mass index (BMI) of 30 kg/m2 and above [1]. Obesity is a common health problem, associated with type 2 diabetes, hypertension, dyslipidemia, and ischemic heart disease [2, 3]. It is reported to be the fifth leading cause of all deaths [2]. In 2022, the prevalence of obesity in Turkiye was estimated as 20.2% of the entire adult population, and 23.6% in women only [4]. Metabolic and bariatric surgeries are currently the most effective treatment methods applied for obesity and related diseases and are reported to be superior to some medical treatment methods. This form of surgery has been shown to provide reductions in BMI and anxiety, as well as improvements in overall quality of life, self-esteem, and sexual function [5]. In terms of mortality, morbidity, cost-effectiveness, patient satisfaction, and excess weight loss rates, laparascopic sleeve gastrectomy (LSG) is one of the most frequently used bariatric surgical procedures [2, 5, 6].

Obesity has been shown to negatively affect the quality of daily life, and menstrual and sexual function [5, 7]. The rate of sexual dysfunction in obese women was found to be 46% [8]. In addition to normalization of the menstrual cycle, an improvement in dysmenorrhea was also observed after LSG [9]. By losing weight, the aim should not only be for patients to regain their health, but also to restore their sexual and menstrual function that have been impaired in some way. Weight loss results differ between genders [3]. Normal female sexual response is complex [10], which may explain why overweight/obese male patients were preferred in most of the few studies conducted on BMI and sexual functionality [2, 5, 6]. In the present study, the aim was to investigate the effects of excess weight loss (EWL) after LSG on female sexual function, frequency of sexual intercourse, menstrual patterns, and menstrual pain, if any.

## Methods

Sexually active female patients who underwent LSG due to obesity between 2016 and 2022, and were attending a University Hospital, General Surgery Clinic were eligible for inclusion. Informed consent was obtained from all individual participants included in the study. This study was approved by the Kocaeli University Faculty of Medicine human ethics committee (KÜ GOKAEK-2024/01.02).

The BMI and EWL of these patients were evaluated by the general surgeon preoperatively and 1 year postoperatively. In addition, a gynecologist investigated complaints of sexual dysfunction, frequency of sexual intercourse, menstruation regularity, and dysmenorrhea before surgery and at the end of the first postoperative year. The Female Sexual Function Scale (FSFI) questionnaire was used to assess sexual functions. The highest possible score in the FSFI survey is 36 and the lowest score is 2. An FSFI score below 26.55 is defined as compatible with sexual dysfunction. Turkish validity and reliability of the FSFI was conducted by Aygın et al. [11]. Since patients who did not have sexual intercourse could not answer some of the questions in the FSFI, only patients who had sexual intercourse before and after surgery were included in the study. The FSFI form was presented to the patients in strictest confidence. However, they could be helped by the gynecologist, if they wanted.

Participants meeting the following criteria were included in the study: (I) sexually active female patients with a partner; (II) without dysfunction in the male partner that prevents him from having sexual intercourse; (III) with a BMI ≥ 35 and comorbid disease or a BMI ˃40 kg/m2; (IV) being on a diet recommended by a dietitian for at least 6 months before surgery; (V) having received approval for LSG from the institutional Bariatric Surgery Council; (VI) planned to undergo LSG; (VII) not having been previously treated with any bariatric surgery; (VIII) with sufficient capacity to answer survey questions; (IX) undiagnosed with endometriosis and/or adenomyosis during gynecological examination; (X) who have regular or irregular menstruation and are not in menopause; and (XI) without type 2 diabetes mellitus (type 2 DM) and/or cardiovascular disease. Patients with type 2 DM and/or cardiovascular diseases were excluded due to the potential to impair sexual function, even if the patients were not obese [12–14].

In addition, another questionnaire about menstruation and dysmenorrhea was presented to the patients. Menstruation regularity was defined as an interval cycle from 21 to 35 days. When questioning menstrual regularity, they were asked whether less than 21 days or more than 45 days had passed between two cycles, whether they had regular menstrual periods with intervals of 21–35 days, and whether it had been 45 days–6 month intervals between menstrual periods.

Dysmenorrhea was evaluated with the Visual Analog Scale (VAS). Pain intensity is evaluated by scoring between 0 and 10. On the scale, < 3 is indicated as mild pain, 3–6 as moderate pain, and > 6 as severe pain. If there was no dysmenorrhea, the score was 0 [15].

Statistical evaluation was performed with SPSS, version 20.0 (IBM Corp., Armonk, NY, USA). Compliance with normal distribution was evaluated with the Kolmogorov–Smirnov test. Variables with normal distribution are given as mean ± standard deviation (SD) and variables with non-normal distribution are given as median and interquartile range (25th–75th percentile). Categorical variables are given as frequency (percentage). The Marginal Homogeneity test was used for dependent group comparisons for categorical variables. Paired t test and Wilcoxon Signed-Rank test were used for dependent group comparisons for numerical variables. Correlation between numerical variables was investigated by Spearman correlation analysis. In hypothesis tests, a *p* < 0.05 was considered sufficient for statistical significance.

## Results

**Fig. 1:**
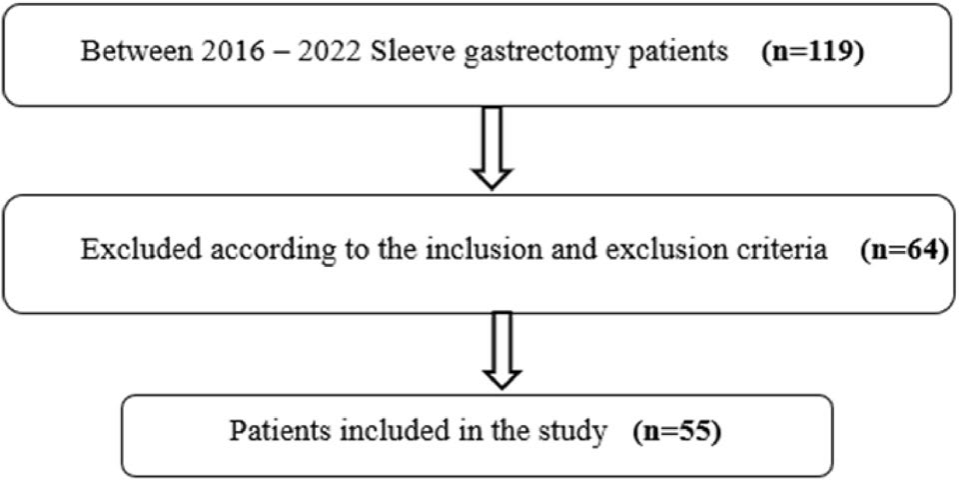
The flowchart of the study

A total of 55 patients took part in the study, with a median (IQR) age of 37 (20–61) years (Fig. 1). All patients diagnosed with thyroid disorders were receiving treatment and were euthyroid at the time of the study. Upon examining socioeconomic levels, it was observed that the majority of patients were classified as having a moderate socioeconomic status (Table 1). The mean ± SD BMI on the day of the operation was 45.32 ± 5.824 kg/m2. The mean BMI value at the end of the first postoperative year was 27.88 ± 1.99 (*p* < 0.001). Furthermore, the mean EWL at the end of 1 year was 73.09 ± 19.74%. The median preoperative FSFI score was 26.3 (22.7–27.4). One year after LSG, this value had significantly improved to 34.5 (30.2–35.3) (*p* < 0.001). One week before surgery, the median frequency of sexual intercourse was 2 (1–3) times per week, and this frequency had significantly increased to 4 (3–4) after surgery (*p* < 0.001) (Table 2).

**Table 1:** Demographic and clinical characteristics of the study population (*n* = 55)

Age (years), median (IQR)	37 (20–61)
BMI (kg/m^2^), mean ± SD	45.32 ± 5.824
Duration of marriage (years), median (IQR)	10 (2–21)
Gravida, median (IQR)	2 (1–3)
Thyroid disorders, *n*(%)	
No	49 (89.1)
Hypothyroidism	4 (7.3)
Hyperthyroidism	2 (3.6)
Socioeconomic level, *n*(%)	
Below minimum wage	5 (9.1)
Middle	47 (85.5)
Above minimum wage	3 (5.4)

^a^*BMI* Body mass index

**Table 2:** Comparison of changes occurring with decreasing BMI between pre- and postoperative periods

	Preoperative	Postoperative first year	p*
BMI^a^ (kg/m^2^)	45.32 (± 5.82)	27.88 (± 1.99)	** < 0.001**
Sexual function			
Median FSFI^b^ score	26.3 (22.7–27.4)	34.5 (30.2–35.3)	** < 0.001**
Sexual intercourse (median n/week)	2 (1.00–3.00)	4 (3.00–4.00)	** < 0.001**
Dysmenorrhea VAS^c^ scores, *n* (%)			
Severe (>6)	0	0	
Moderate (3–6)	5 (9.1%)	0	0.819
Mild (< 3)	17 (30.9%)	28 (50.9%)	
Negative (0)	33 (60%)	27 (49.1%)	
Menstrual frequent periods, *n* (%)			
Short Periods (< 21 days)	6 (10.9%)	0	
Long Periods (> 45 days)	18 (32.7%)	6 (10.9%)	** < 0.001**
Regular (21–35 days)	19 (34.5%)	39 (70.9%)	
No menstruation (45 days–6 months)	12 (21.8%)	10 (18.2%)	

^a^MI: Body mass index

^b^FSFI: Female sexual function index

^c^VAS: Visual Analog Scale

*p value: *p* < 0.05 significant

Table 2 also shows a comparison of the menstrual pattern before and after surgery. While the number of patients with menstrual periods longer than 45 days was 17 (30.9%), this number decreased to 6 (10.9%) at the end of the first postoperative year. Moreover, the number of patients whose menstrual period was shorter than 21 days was 6 (10.9%) preoperatively, but all had a normal menstruation period after surgery. The number of patients with completely normal menstrual patterns increased from 19 (34.5%) in the preoperative period to 39 (70.9%) one year after LSG. Of the 12 (21.8%) patients who had not had a menstrual period for 6 months preoperatively, two had regular menstruation postoperatively (*p* < 0.001).

VAS scores are also given in Table 2. There were no patients with severe dysmenorrhea. While there were five (9.1%) patients who experienced moderate dysmenorrhea in the preoperative period, moderate dysmenorrhea was not detected in any patient in the postoperative period. The proportion of patients experiencing mild menstrual pain (*n* = 17, 30.9%) increased to 28 (50.9%) after 1 year. While there were 33 (60%) patients who did not experience menstrual pain preoperatively, no menstrual pain was reported by 27 (49.1%) at the evaluation made 1 year after the surgery. However, this change in proportion was not significant (*p* = 0.819).

## Discussion

Sexual dysfunction negatively affects self-esteem, quality of life, and the relationship between sexual partners [12]. Female orgasm, as a component of sexual function, is influenced by various factors, including communication with a sexual partner, emotional closeness, male sexual performance, chronic pain, as well as factors such as body image and self-esteem [16]. Various causes of sexual dysfunction are the most common reason for visits to the gynecology outpatient clinic. Obesity is an increasingly common public health problem that has been shown to reduce quality of life and impair sexual functionality [5, 7]. Furthermore, obese women often present with oligomenorrhea, amenorrhea, or irregular menstrual periods [17]. It was reported that FSFI scores were decreased in patients with irregular menstruation, regardless of age, BMI, socioeconomic status, or parity [12]. Cyclic dydrogesterone treatment, by correcting bleeding disorders, has been shown to improve sexual function, including desire, arousal, and lubrication, more effectively than LNG-IUD [18]. Thus, it was shown that obesity may cause sexual dysfunction directly or indirectly by causing menstrual complaints. Although sexual dysfunction was initially defined as a psychological disorder, it is nowadays accepted that sexual dysfunction is a multifactorial condition with neurobiological, hormonal, and psychosocial aspects [5].

The locus of almost all sexual responses are in the brain, and therefore psychosocial and/or behavioral and/or developmental, and cultural factors play a central role in sexual well-being. Normal female sexual response may be mediated by many well-known neurotransmitters, but especially dopamine, and is strongly influenced by steroid hormones [10]. In addition, measuring the levels of steroid hormones, including follicle stimulating hormone or estrogen, for example, or neurotransmitters would have allowed for an analysis of the changes in their levels associated with sexual dysfunctions. Since the present study was retrospective, these data were unfortunately unavailable. As noted, the underlying causes of sexual dysfunction are multifactorial. However, it is important to treat at least one of these factors, obesity [7]. Treating obesity may improve or resolve sexual dysfunction [3].

Obesity surgery is offered to patients with obesity with a BMI of ≥ 40 kg/m^2^ or patients with a BMI of ≥ 35 kg/m2 ^2^ith accompanying comorbidities [2]. The preoperative BMI values in the present study were within these limits. After LSG, the clinical aim is to achieve a BMI below 30 kg/m^2^ [19]. The BMI values of patients in the present study decreased to a median of approximately 28 kg/m^2^. Moreover, the mean percentage decrease in EWL at the end of 1 year was around 73%. While restoring the health of female patients with obesity surgery, the clinical aim should also include restoration of any impaired sexual function [20].

Although there are many studies into obesity, its consequences in terms of sexuality are less well understood. In addition, sexual dysfunction is more commonly neglected in women, possibly due to stigma and taboo [13]. We investigated the sexual function of female patients who underwent LSG, had significant decrease in BMI and a substantial EWL% within 1 year of surgery, using the FSFI questionnaire. In a study conducted on male patients, improvement in sexual function, increase in satisfaction, and even improvement in fertility were detected with the decrease in BMI after bariatric surgery [2].

The median preoperative FSFI scores of patients was 26.3 and was considered to indicate sexual dysfunction, even though this value was just below the cut-off value cited for the FSFI scoring system. One year after the LSG, the median FSFI score was 34.5, indicating no sexual dysfunction and was also significantly improved compared to the baseline.

Frequency of sexual activity is also important [21]. Any change in the frequency of sexual intercourse following LSG was investigated in the present study. One week before surgery, the median frequency of sexual intercourses was twice per week, but had increased to four times per week 1 year after surgery (*p* < 0.001). Thus, both the FSFI scores and the frequency of sexual intercourse had significantly increased postoperatively.

After successful sleeve gastrectomy surgery, improvement in menstrual and ovarian function along with improved biomarkers of metabolic syndrome has been previously reported [6, 9, 17]. Pilone et al. observed an improvement in the menstrual cycle and resolution of dysmenorrhea after LSG [9]. Since it is known that ovarian function generally improves after excess weight loss and ovulation occurs more regularly [3]. one of our aims was to investigate any changes in self-reported dysmenorrhea, as it occurs more frequently with ovulation. Most of the patients who attend our outpatient clinic after weight gain or weight loss have been asked about dysmenorrhea and menstrual changes. Although there are many causes of dysmenorrhea, we wanted to investigate it in the context of pre- and postoperation LSG in the study cohort. For this reason, patients suspected of having endometriosis and/or adenomyosis were not included in the study. However, no significant difference was observed when the severity of dysmenorrhea and the number of patients suffering from it were evaluated.

Primary dysmenorrhea occurs as a result of myometrial contractions and ischemia after ovulation [22]. According to some studies, a significant improvement in ovulation rates was observed when excess weight was lost [23, 24]. Postoperative dysmenorhea was observed in only six patients without dysmenorrhea preoperatively. Four of these six patients had menstruation approximately once every 45 days before surgery, while the other two had spotty and irregular menstruation. Of note, the menstrual cycles of all six patients were regularized after surgery. This suggested that ovarian function improved with EWL. A statistically significant decrease was detected in patients experiencing menstrual irregularities. However, more robust findings in terms of dysmenorrhea and menstrual function may be obtained if studies can be conducted with larger cohorts.

## Study limitations

There are some limitations of the present study. Although the number of patients was sufficient to draw conclusions, the results would have been statistically more robust with a larger sample size of patients undergoing LSG. In addition, measuring the levels of steroid hormones or neurotransmitters would have allowed for an analysis of the changes in their levels associated with sexual dysfunction. However, since this was a retrospective study, the hormone profiles of all patients could not be accessed. Conditions, such as dysmenorrhea and sexual dysfunction-causing endometriosis and type 2 diabetes, were excluded from our study to evaluate the effectiveness of BMI reduction. However, in another study where the effects of these conditions on sexual and menstrual functions are assessed, meaningful results could be obtained. We noticed that the patients participating in the study were very willing to share the experiences they remembered. However, conducting these studies prospectively will minimize possible self-reporting errors due to a prolonged duration between occurrence and data collection.

It has been reported that obesity reduces sexual function directly or indirectly and FSFI scores increase after EWL. Improving sexual dysfunction is important, so that female sexual and reproductive freedom should be supported by medical providers [20]. Our findings suggest that metabolic and bariatric surgery may be recommended to obese women for sexual dysfunction and/or menstrual problems. Improvement in sexual function may be related to excess weight loss and/or changes in hormone profiles. This may be elucidated through larger prospective studies using qualitative values. Thus, future large prospective studies are needed to understand how sexual and menstrual functions change with significant weight loss.
